# Experimental Study on Tin Slag Polymer Concrete Strengthening under Compression with Metallic Material Confinement

**DOI:** 10.3390/polym15040817

**Published:** 2023-02-06

**Authors:** Muhamad Soffi Manda, Mohd Ruzaimi Mat Rejab, Shukur Abu Hassan, Mat Uzir Wahit, Didik Nurhadiyanto

**Affiliations:** 1 Structural Performance Material Engineering (SUPREME), Faculty of Mechanical & Automotive Engineering Technology, Universiti Malaysia Pahang, Pekan 26600, Pahang, Malaysia; 2 Department of Mechanical Engineering, Polytechnic Sultan Haji Ahmad Shah (POLISAS), Semambu, Kuantan 25350, Pahang, Malaysia; 3 School of Chemical and Energy Engineering, Faculty of Engineering, Universiti Teknologi Malaysia (UTM), Skudai, Johor Bahru 81310, Johor, Malaysia; 4 Centre for Advanced Composites (CACM), Universiti Teknologi Malaysia (UTM), Skudai, Johor Bahru 81310, Johor, Malaysia; 5 Department of Mechanical Engineering Education, Faculty of Engineering, Universitas Negeri Yogyakarta, Yogyakarta 55281, Indonesia

**Keywords:** TSPC, strengthening, confinement, metal, compression, stress, strain

## Abstract

Studies on the external strengthening of tin slag polymer concrete by fibre-reinforced plastic confinement have provided strength enhancement of tin slag polymer concrete up to 128% with carbon fibre-reinforced plastic confinement. However, the effect of metallic material confinement has yet to be studied. This article presents the experimental finding on tin slag polymer concrete strengthening through metallic material confinement under compressive loads. Machined mild steel metal tube has been employed to strengthen tin slag polymer concrete core in partial and fully confinement prior to compression testing. Through this study, compressive strength of tin slag polymer concrete short column has been enhanced with the metal tube confinement application from 59.19 MPa (unconfined) to 95.86 MPa (partial metal confinement) and 131.84 (full metal confinement) representing 61.95% and 122.74% of strength enhancement percentage. Material behaviour analysis through stress versus strain curves has revealed that the strain softening curve is modified by metal tube confinement before a fracture occurs on both partial and full metal confinement samples compared to the control sample (unconfined). In addition, the failure modes have indicated that the high ductility of metallic confinement material has effectively confined tin slag polymer concrete from sudden fracture where the metal tube in partial confinement indicates ductile expansion while the metal tube in full confinement has shown ductile crushing. In general, it was concluded that metallic material confinement on tin slag polymer concrete under compressive load has resulted in providing strength enhancement and modified the failure mode of tin slag polymer concrete. Finally, further research is recommended, especially by initiating numerical analysis to facilitate parametric studies on tin slag polymer concrete for structural material design.

## 1. Introduction

Polymer concrete (PC) is an alternative to cement concrete material which employs polymeric resin as a matrix binder together with conventional aggregates primarily of sands and gravels. According to Mebarkia et al. [1], PC poses a wider range of strength with 40–60 MPa compared to cement concrete strength range of 10–60 MPa. In addition to that, PC has good practicability due to the various types of polymeric resins available to be applied as matrix elements and also has high compatibility with the consumption of alternative aggregates, especially from waste and slag materials [2,3,4,5]. Recently, a by-product of the tin smelting process has been introduced as aggregates in polyester-based polymer concrete. Tin slag polymer concrete (TSPC) is one of the particle-reinforced composite materials which consists of tin slag (TS) particles as reinforcement and polymeric resin as a matrix. TSPC study has made a debut by evaluating on its potential as alternative concrete material to be employed in structural material application. According to Faidzal et al. [6], TSPC was able to produce the optimum compressive strength (58 MPa) by 70:30 aggregate-to-resin ratio with uniformly graded fine TS particles (<1 mm) mixed with unsaturated polyester resin (UPR) that has been polymerized by methyl ethyl ketone peroxide (MEKP) with 2% of the resin weight. The wet mixture was then has been cast into a 50 mm diameter specimen mould with 100 mm height and cured at room temperature for 3 days prior to compression testing. The superiority of TSPC over conventional cement concrete material originated from the preferable properties of polymer concrete and the fact that TSPC is produced by recycling industrial waste as its primary composition. According to previous studies, polymer concrete is superior to cement concrete in terms of strength, quick curing, low cured shrinkage, excellent adhesion to most surfaces, resistance to chemicals and corrosion, excellent damping properties, low water absorbability, and the ability to be cast into complex shapes [7,8,9,10]. In addition to that, TSPC in particular has applied TS waste as aggregates which provide a solution to tin smelting production and also preserve natural aggregates. Reducing the dependency on natural aggregates is becoming essential for sustainability goals as natural aggregates, such as gravels, granites, stones and river sands are widely used in concrete production, and the consumption of these aggregates has caused the increasing trend in the activity of the aggregate mining process [11]. According to Ozcan et al. [12] and Ukpong [13], the effects of natural aggregate mining are water pollution, sediment growth, mudflows, erosions, landslides or noise and dust due to stone blasting effects. Therefore, by promoting TS particles as aggregates, TSPC may preserve the consumption of natural aggregates and thus sustain the resources.

A recent trend in TSPC studies since Faidzal et al. [6] has been dominated by studies on the strengthening potential of the TSPC under compressive loading through external means. According to Raza et al. [14], external strengthening of concrete structures has become popular compared to traditional strengthening due to ease and speed of installation, less labour work, minimum change to the original geometry and aesthetics of the structure, high strength-to-weight ratio, and most importantly, its occupant-friendly nature (repair on site). Strengthening is a process to enhance the mechanical properties of a material, especially those designed for structural application. According to Ueda [15], the durability of structural material is a top priority because structural material in service will face natural disasters or unexpected destructive forces from earthquakes, tsunamis, tornados, cyclic loadings, creeps, and chemical or frost attacks. Therefore, strengthening structural materials may provide safety, cost savings and prolong material service life. In the case of TSPC, studies on external strengthening by glass fibre-reinforced polymer (GFRP) and carbon fibre-reinforced polymer (CFRP) confinement with various parameters have been performed [16,17,18,19]. To summarise, the study revealed that the highest TSPC strength enhancement has occurred with one layer of CFRP wrapping with up to 128% compared to unconfined TSPC. In addition to strength enhancement, cement concrete material generally failed with brittle shear failure due to crushing during compressive loading application [20]. However, according to Manda et al. [7], TSPC has shown brittle failure with additional plastic straining behaviour and the addition of FRP confinement has further modified the fracture behaviour of TSPC through effective stress transfer from the TSPC core to confinement material.

Although FRP confinement is proven capable of producing an enhanced strength of concrete column advantages to its strength-to-weight ratio behaviour, the failure mode of FRP confined concrete is explosive and sudden with no warning sign due to its brittle inheritance nature of FRP in tension which is undesirable in design consideration. Therefore, Chin et al. [21], suggested steel tube confinement is safer to manipulate the ductile property of steel so that the failure will occur gradually with pre-failure warning signs which are more desirable in the design consideration of structural material. According to Zhou et al. [22] and Kedziora et al. [23], when a steel tube jacketing is used to externally strengthen a concrete column, the brittle shear failure was effectively prevented with the increase in lateral strength resulting in higher compressive strength compared to an unconfined concrete column. The study reported that there was very little effect observed on plastic deformation capacity, indicating that the metal tube confinement has provided enough ductility to reduce the brittleness of the core concrete column during crushing failure. The effectiveness of metallic material jacketing has been proven to enhance the strength and behaviour of conventional concrete material but for TSPC, a study on metallic material confinement has yet to be found in the literature. Therefore, the purpose of this article is to present the experimental finding on TSPC strengthening under compressive loading with metallic material confinement. The finding will open up space for extensive studies in the application of metallic material confinement on TSPC especially in exploring its strengthening potential for structural material design application.

## 2. Materials and Methods

### 2.1. TSPC Core Specimen

The whole test specimen consists of two parts: the TSPC core as main column specimen and the machined mild steel (MS) tube as metallic material confinement to strengthen the TSPC core. The main reference in fabricating the TSPC specimen is according to previous work completed by Faidzal et al. [6], Shakil and Hassan [16], Hassan et al. [17], Amirnuddin et al. [19], Abdullah [18] and Manda et al. [7]. In this research, TSPC cylindrical column specimens were prepared with casting process using 50 mm diameter and 100 mm length PVC pipe as mould. During the wet mixture, TSPC composition consists of fine TS particles (<1 mm) as aggregates and UPR as matrix binder with a ratio of 70:30. This composition is selected based on optimum performance of TSPC from the literature. Table 1 presents the performance of TSPC with different composition proportions and preparation methods from previous studies.

**Figure 1:**
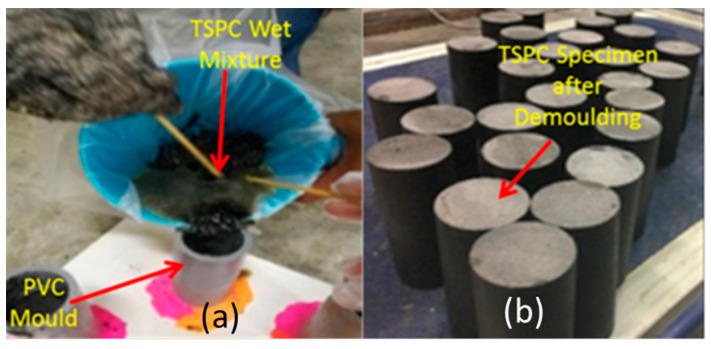
TSPC column specimen preparation. (**a**) Casting wet mixture of TSPC into specimen mould. (**b**) TSPC specimen after demoulding.

Before being ready to be employed as binder in the wet mixture, the UPR is mixed with methyl ethyl ketone peroxide (MEKP) as hardening agent with 2% from total liquid resin weight according to resin manufacturer’s recommendation. Before demoulding, the TSPC was cured for minimum of 3 days at room temperature. The guideline for TSPC specimen preparation is based on ASTM C192/470 standard specifications for making and curing concrete test specimens and moulds for forming concrete test cylinders vertically [24,25]. Figure 1 shows the process of TSPC column specimen preparation.

### 2.2. Metallic Material Confinement

**Figure 2:**
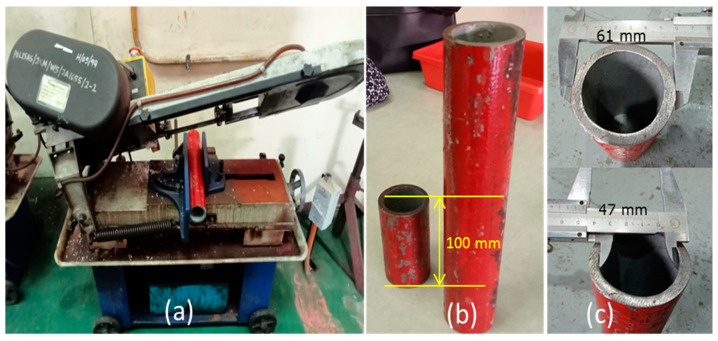
Preliminary process of metal tube confinement fabrication. (**a**) Cutting the metal pipe using bench saw. (**b**) Metal tube with 100 mm length representing TSPC specimen height. (**c**) The initial OD and ID of the metal tube resulting in 7 mm thickness.

Metallic material confinement is applied in this study to strengthen the TSPC column under compressive load. Steel tube is applied for the metallic material confinement and selection of the steel tube is based on Umamaheswari et al. [26], which uses cold-formed steel hollow. Specifically, the metallic material employed is ASTM 106 MS Pipe 61 mm outside diameter (OD) × 47 mm inside diameter (ID). The pipe was originally in 330 mm length. To fabricate the metal tube confinement for TSPC, the pipe was cut into 100 mm length using a bench saw machine which is equivalent to TSPC column specimen height. Figure 2 shows the preliminary process of fabricating the metal tube confinement.

**Figure 3:**
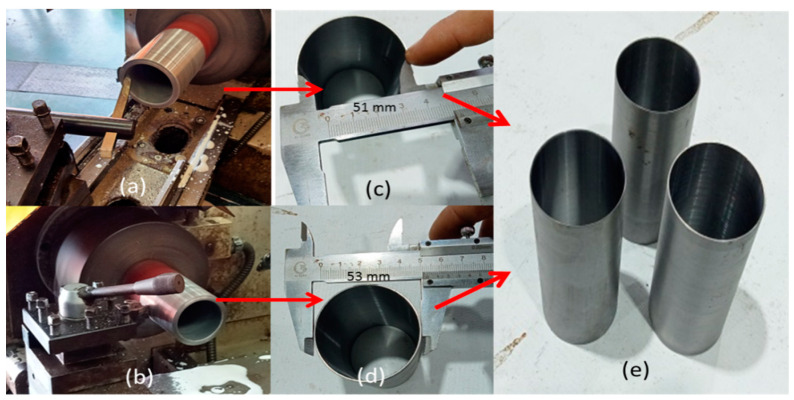
Machining process on MS pipe to make metal tube confinement on TSPC specimen. (**a**) Internal surface lathe (turning). (**b**) External surface lathe (turning). (**c**) Internal diameter increased from 49 mm to 51 mm. (**d**) Outside diameter decreased from 61 mm to 53 mm. (**e**) The finished and metal tube confinement (1 mm thickness).

Further process was performed to confine the 50 mm diameter TSPC column where the 100 mm MS pipe is machined by internal surface turning to achieve 51 mm internal diameter preparing for 0.5 mm thickness for adhesive material. Then, the MS pipe’s external surface turns until it achieves a 53 mm diameter to provide metallic material confinement thickness of 1.0 mm on the TSPC column. Figure 3 shows the 100 mm MS pipe machining process of 100 mm MS pipe machining to achieve the desired dimension as metal tube confinement material on TSPC column specimen.

**Figure 4:**
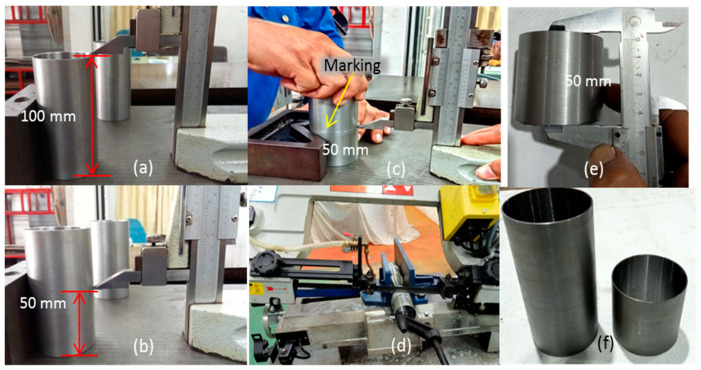
Metal tube preparation for TSPC confinement. (**a**) The 100 mm metal tube height. (**b**) The 50 mm measurement for partial metal tube confinement. (**c**) Marking on 50 mm for cutting preparation. (**d**) Cutting the metal tube in half. (**e**) Finished preparing 50 mm height metal tube. (**f**) Ready metal tube for full and partial confinement process.

This study’s variation of metallic material confinement is divided into unconfined TSPC for control test, 50 mm height MS tube for partial metallic material confinement and 100 mm height for full confinement on the TSPC column. After the machining process has achieved the designed parameter, the MS tube is pre-treated where the tube internal surface is cleaned using sand paper and solvent cleaner to remove dirt, grease, oil, and oxides. This process is important to allow efficient bond between the MS tube and TSPC core using the adhesive material. Figure 4 shows the metal tube preparation to provide variations in metallic material confinement on TSPC column specimen by partial and full confinement.

### 2.3. TSPC with Metallic Material Confinement

**Figure 5:**
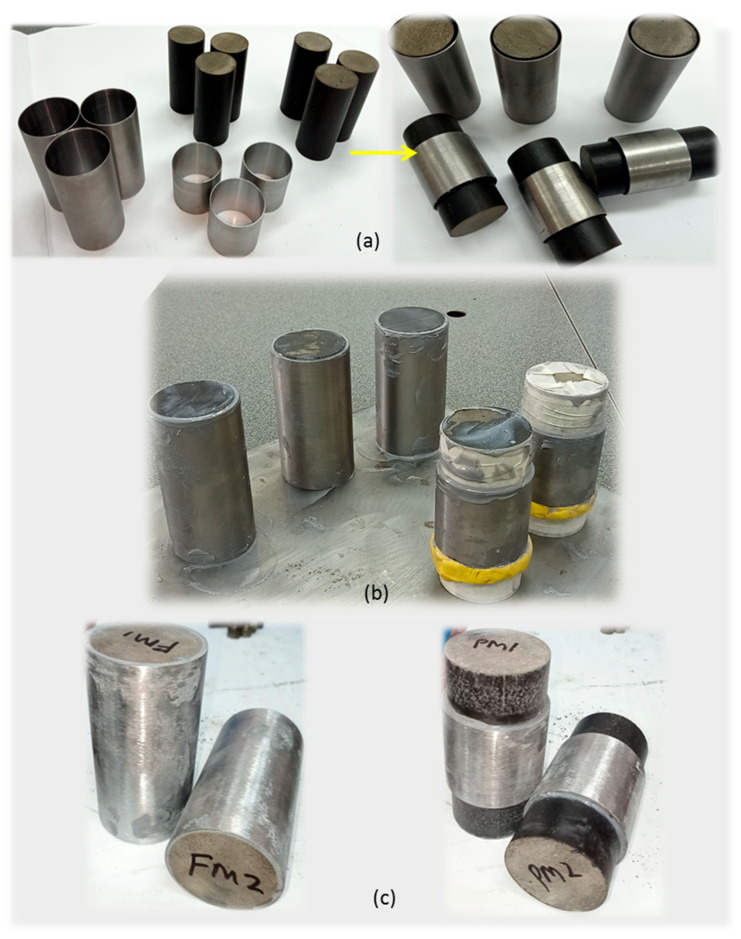
Specimen fabrication process. (**a**) Metal tube ready to confine the TSPC column. (**b**) Application of Sikadur 330 adhesive and curing of TSPC with metal tube confinement (binder thickness 0.5–1.0 mm). (**c**) Finished samples consist of TSPC with full and partial metal tube confinement specimens.

After the confinement material is ready, additional process for sample fabrication is to install the confinement on TSPC column using adhesive. The adhesive employed is Epoxy Sikadur 330-part A and part B from Sika AG. Before the confinement is performed, 6 units of TSPC specimens are cleaned on their external surface using acetone and let dry. The process started with mixing Sikadur 330 by 56 g part A and 14 g part B = 70 g (4:1) into a container and stirred well. After that, the epoxy mixture was applied on both surface of TSPC and metal confinement. Then, the ready metal tube was sleeved into TSPC each in 3 units for full confinement (100 mm) and 3 units for partial confinement (50 mm). Figure 5 shows the entire specimen fabrication process. The external strengthening of TSPC specimen has been performed according to ACI 440.2R-08 guide for the design and construction of externally bonded confinement systems for strengthening concrete structures [27].

### 2.4. Test Sample Specification

All specimens are classified as TSPC column control samples (unconfined), TSPC with partial metal confinement and TSPC with full metal confinement. The specimen is coded as UC for control sample, PM for partial confinement sample and FM for full confinement sample. Each sample is provided with three similar specimens (UC1, UC2, UC3; PM1, PM2, PM3; FM1, FM2, FM3) to validate the results through data comparison by observation in repetitive testing outcomes. Partial confinement is located at the middle section of TSPC column as a previous study reveals that most TSPC failed under crushing failure at the middle section in addition to some shear failure. The epoxy adhesive and confinement material thickness range from 1.5 mm to 2.0 mm. The curing time for confinement is 30 days as recommended by adhesive Epoxy Sikadur 330 manufacturer. Table 2 presents the description of each sample and specimen code.

### 2.5. Mechanical Testing

**Figure 6:**
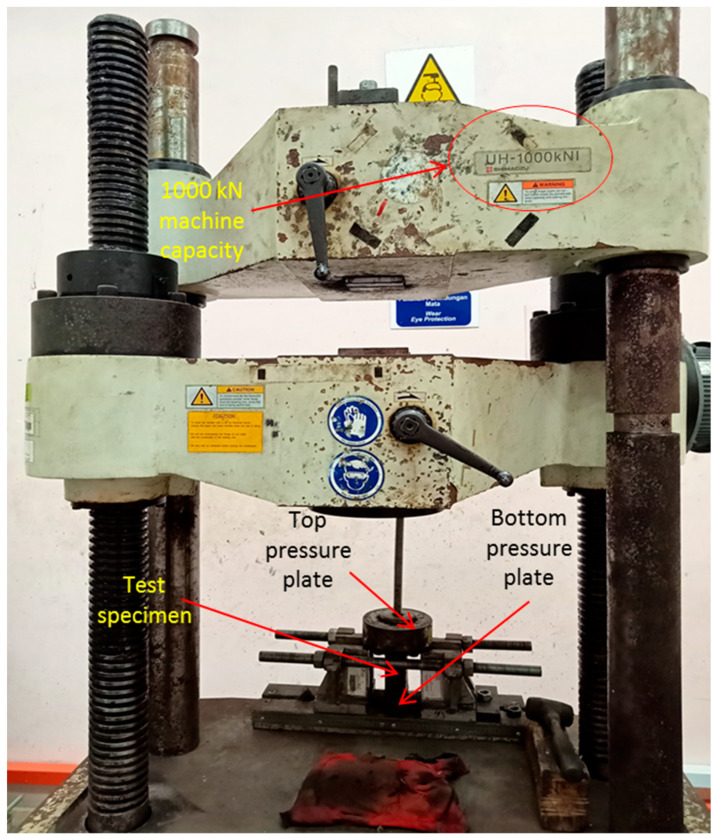
Mechanical test set-up.

The mechanical testing set up is based on previous study on TSPC compressive behaviour by Faidzal et al. [6], Shakil and Hassan [16], Hassan et al. [17], and Amirnuddin et al. [19]. Uniaxial compression test is applied on the test sample and the mechanical testing is performed using Shimadzu 1000 kN Universal Testing Machine. Test sample is located carefully on the middle section of the bottom pressure plate to avoid buckling failure due to sample distortion upon placement. The compressive load is exerted on the test sample by the top pressure plate of the testing machine. A digital camera is also placed beside the testing machine to facilitate the failure mechanism analysis after the testing process. The loading rate of the compressive load was 1 mm/ min. The test sample designations were as previously described: UC for unconfined TSPC, PM for TSPC with partial metal tube confinement and FM for TSPC with full metal tube confinement. In general, the mechanical testing procedure was performed based on ASTM C 579-01 standard test methods for compressive strength of polymer concretes [28]. Figure 6 shows the mechanical test set-up.

## 3. Results and Discussions

### 3.1. Average Compressive Strength

The outcome of mechanical testing has measured several data for each test specimen, including maximum deformation, maximum load, compressive strength, and fractured energy. In addition to that, the average compressive strength for PM and FM sample is also calculated together with the percentage of strength enhancement compared to the unconfined TSPC sample (UC). Table 3 presents the experimental testing results for all of the test samples (UC, PM and FM) consisting of nine specimens, namely UC1, UC2, UC3, PM1, PM2, PM3, FM1, FM2, and FM3. There was the highest level of consistency in the test results for sample UC where for all of the specimen (UC1, UC2 and UC3), the experimental result has provided measurement in smallest deviation for all of the deformation, loads, compressive strength, and fractured energy value. The deformation for the UC sample is between 3.028–3.07 mm corresponding to maximum load with a fall within 115 kN–116.22 kN. The compressive strength measurement ranges from 57.34 MPa to 59.19 MPa and the fracture energy for UC samples is in between 341.39 J to 387.01 J. The average compressive strength for unconfined TSPC (UC sample) is 58.37 MPa and as a control sample, the data for percentage of strength enhancement is not required for this sample.

Then, for sample PM, test results revealed different findings with larger deviations among data measured except for load and compressive strength for PM2 and PM3 which is considered acceptable in terms of consistency. The experimental results on PM1 have indicated a potential for excessive pre-loading compared with the results of PM2 and PM3. The deformation for each specimen (PM1, PM2, and PM3) is 2.614 mm, 4.288 mm, and 3.458 mm corresponding to maximum loads of 188.23 kN, 155.28 kN, and 154.44 kN. The compressive strength and fracture energy measurement for sample PM is also in a similar pattern with maximum loads of 95.86 MPa, 79.08 MPa, and 78.66 MPa (PM1, PM2, and PM3), while fracture energy is 1123.74 J, 1080.3 J, and 960.23 J (PM1, PM2, and PM3). The fracture energy value indicates that PM1 has to absorb larger energy (1123.74 J) to cause fracture compared with PM2 (1080.3 J) and PM3 (960.23 J). The average compressive strength for TSPC with partial metal tube confinement is 84.53 MPa. Calculation on compressive strength enhancement after the TSPC column specimen is strengthened with partial metal tube confinement results in 44.83% of increments compared to unconfined TSPC as the control test specimen. The percentage of strength enhancement of the highest strength is on specimen PM1 (95.86 MPa) from UC1 (59.19 MPa) with a 61.95% enhancement percentage.

The test results for sample FM indicate the highest deviation, especially for FM3 in terms of deformation. FM3 has achieved 8.986 mm deformation while others are only 2.872 mm and 2.726 mm (FM1 and FM2). However, the maximum load achievement for FM1 and FM2 is larger with 258.86 MPa and 261.52 MPa. These conditions need to be further analysed as obviously, the measurement has revealed that even if FM3 has the highest deformation (8.986 mm), the maximum load measured is only 204.63 MPa indicating that the strain of FM3 has increased without strength enhancement. Then, the compressive strength for sample FM has shown that FM1 has the highest strength with 131.84 MPa followed by FM 2 and FM 3 with 110.27 MPa and 104.22 MPa. However, the fracture energy of sample FM has shown an opposite condition where FM3 has the highest fracture energy followed by FM2 and FM1 with 1821.98 J, 1405.21 J, and 1236.38 J. The average compressive strength for TSPC with fully metal tube confinement is 115.44 MPa. Calculation on compressive strength enhancement after the TSPC column specimen is strengthened with fully metal tube confinement results in 97.78% compared to unconfined TSPC as the control test specimen. The percentage of strength enhancement of the highest strength is on specimen FM1 (131.84 MPa) from UC1 (59.19 MPa) with a 122.74% enhancement percentage.

### 3.2. Load versus Deformation

**Figure 7:**
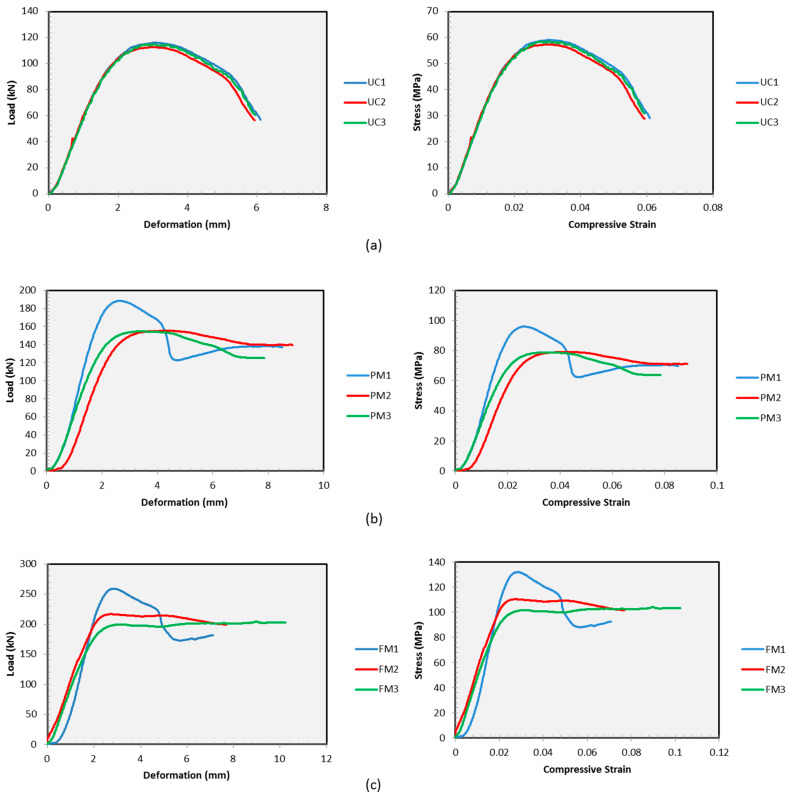
Load/stress versus deformation/strain curve. (**a**) Unconfined TSPC (UC). (**b**) TSPC with partial metal tube confinement (PM). (**c**) TSPC with fully metal tube confinement (FM).

The load versus deformation curve represents the deformation that occurs for every step of load increment on a material based on a specified loading rate. The material then resisted the deformation based on its cross-sectional area and the level of this resistance is known as the material strength which also causes strain based on the ratio of the change in shape that occurs to the original shape of the material. Therefore, other than load versus deformation, another material behaviour curve which is the stress versus strain curve must be observed to analyse the material behaviour under external load application. In nature, under load application, all materials are elastic up to a certain extent. To this extent, material behaviour will exhibit a linear relationship and the slope of the linear curve representing the stiffness of the material, known as Young’s modulus. Then, the material will yield before experiencing plastic behaviour which represents by a more complex and nonlinear relationship up to fracture. Figure 7a shows that the control sample (UC) has shown consistency for every specimen in both curves (load–deformation and stress–strain). During initial loading, the UC sample exhibits a linear relationship up to a yield point and then turns into strain hardening up to maximum load. After that, the UC samples start to soften until they fracture. Then, the length of softening curve indicates that the unconfined TSPC has gradually produced crack initiation before the crack propagates until actually fractured. Other than crack, shear failure or crushing failure may also be expected to occur with the same mechanism where it initiates and propagates before fracture. From the curves, it is expected that TSPC is not totally brittle as the failure does not occur in a sudden manner as most cement concrete material.

Figure 7b shows the material behaviour for TSPC with partial metal tube confinement (PM sample) under compressive loadings. Initially, all PM specimens exhibited linear behaviour (elastic) up to the yield point. After yielding, all of the PM specimens (PM1, PM2, and PM3) again experience strain hardening where the strength continues to increase up to maximum load. Then, upon reaching maximum load, PM1 shows softening behaviour up to a certain extent and regains strength until a fracture occurs during secondary strain hardening behaviour. Different from PM1, the other specimens which are PM2 and PM3 have shown no sign of increase or decrease in strength until fracture occurred. The highest maximum strength is achieved by PM1 which shows the highest peak of the material behaviour curve. Another test sample is FM which described the behaviour of TSPC with fully metal tube confinement under compressive loading. Figure 7c shows that the FM behaviour has shown an almost similar trend to PM behaviour where at first all of the specimens (FM1, FM2, and FM3) experienced linear relationship (elastic) up to yielding. After yielding, all of the FM specimens again experience strain hardening and after reaching maximum strength, FM1 softens before regaining strength and fractures during secondary strain hardening. However, FM2 and FM3 continue to undergo plastic straining without any change in strength magnitude up to fracture. The highest maximum strength is achieved by FM1 which shows the highest peak of material behaviour curve.

### 3.3. Comparison of All Test Sample Behaviour under Compressive Load

The previous section reported the experimental material behaviour for each of the test samples (UC, PM, and FM) separately. In this section, all of the material behaviour curves including the load versus deformation and stress versus strain curve are plotted on the same graph. The reasons are to facilitate observation behaviour and compare the test sample behaviour under compressive load. Through this section, the effect of partial metal tube confinement and fully metal tube confinement on the TSPC column sample under compression may be observed and compared by referring to the material behaviour curve.

**Figure 8:**
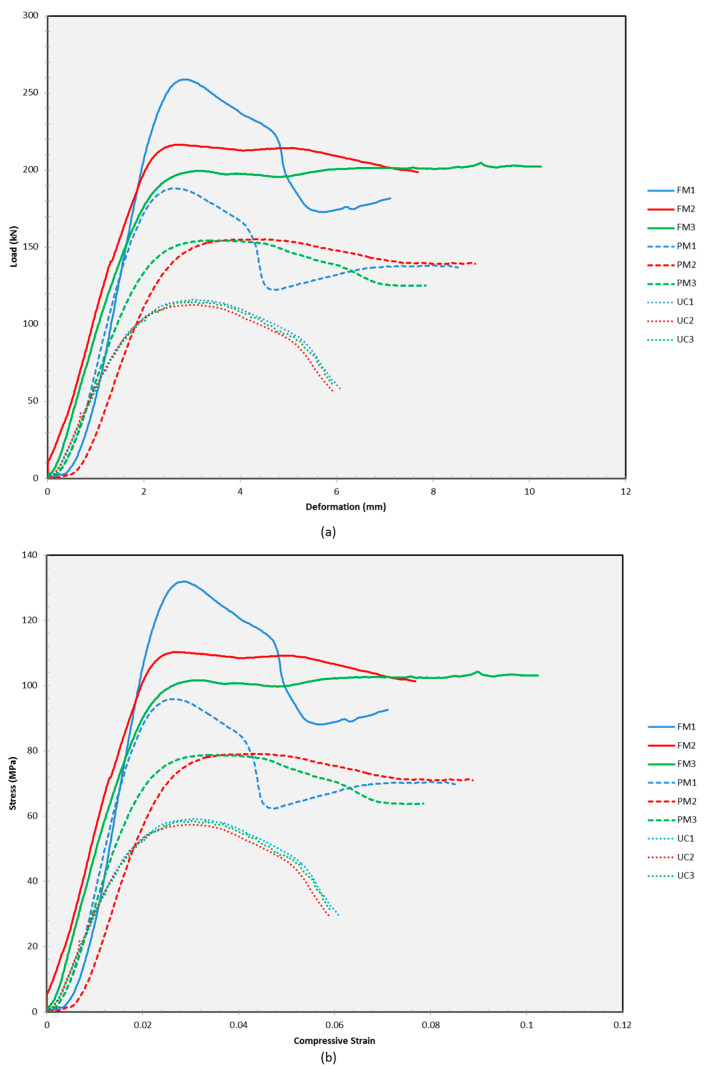
(**a**) Load versus deformation curve for all samples. (**b**) Stress versus strain curve for all samples.

Figure 8 shows the load versus deformation and stress versus strain curve for all of the test specimens from samples UC, PM, and FM. The plotting facilitates detailed comparison among the entire variant of the test specimens. In terms of the curve shape, the UC sample exhibits a simple curve shape compared to PM and FM samples. The reason is that brittle materials such as TSPC always behave in a much simpler response to external load applications similar to other concrete materials. The UC curve has a linear response in the beginning and after yielding, it starts to undergo plastic straining with strain hardening behaviour where the UC sample gains strength with larger strain progression. After maximum loads, the UC sample plastic behaviour exchanges from hardening to softening before a fracture occurs. All UC specimens (UC1, UC2, and UC3) have shown similar behaviour under compressive loads. With the application of partial metal tube confinement, it can be seen that the curve has been modified and exhibit some complexity after the yield point. This condition occurs because metal is a ductile material that responds to complex testing forces, especially under compressive loads. The PM1 specimen has provided higher strength enhancement compared to PM2 and PM3. After yielding, PM1 undergo strain softening behaviour up to a certain extent and regains strength through strain hardening for a while before fracture. PM2 and PM3 have shown almost no hardening or softening after yielding up to a fracture. In comparison with the unconfined specimen, the application of partial metallic material confinement by a metal tube has further enhanced the maximum strength and modified the failure pattern of TSPC.

Then, the application of fully metal tube confinement has provided a similar response with partial confinement. From the curve in Figure 8, FM1 achieved higher strength enhancement compared to PM1 but the material behaviour has shown almost identical response where FM1 also undergo strain softening behaviour up to a certain extent and regain strength through strain hardening for a while before fracture. In addition to that, FM2 and FM3 also have shown almost similar behaviour with PM2 and PM3. Both also exhibit no hardening or softening behaviour after yielding up to fracture. This condition indicates that metal tube confinement either partially or fully modified TSPC behaviour by exhibiting balance in brittle and ductile failure of TSPC. As a comparison, the curves in Figure 8 show that fully metal tube confinement has further enhanced the maximum strength of TSPC compared to partial metal tube confinement on TSPC specimen. In addition, the material behaviour has also exhibited similarity in their response on compressive loads.

### 3.4. Compressive Modulus, Yield Point and Maximum Strength

**Figure 9:**
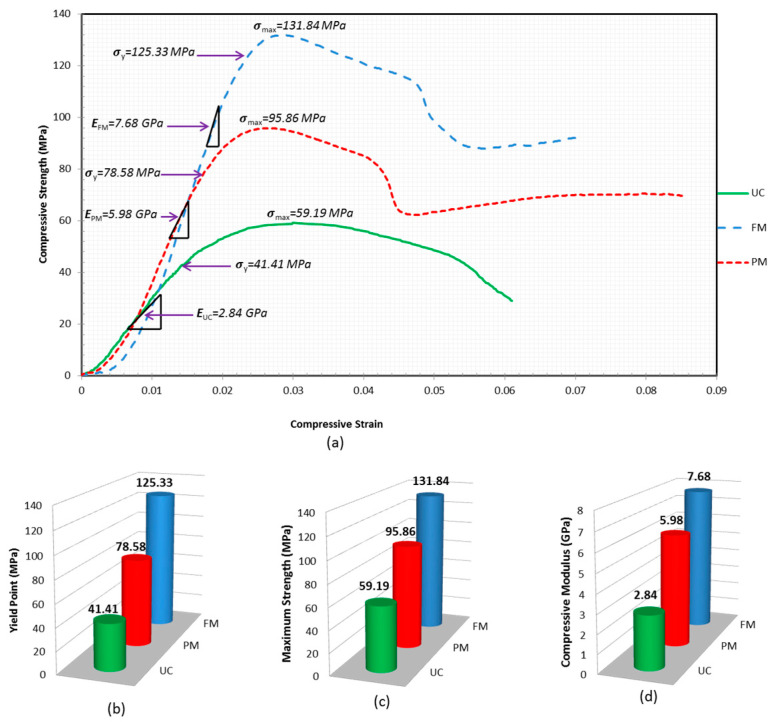
Compressive modulus, yield point, and maximum strength of every test sample. (**a**) Stress versus strain diagram of test samples with maximum strength. (**b**) Bar chart for compressive modulus value. (**c**) Bar chart for yield stress value. (**d**) Bar chart for maximum stress value.

The experimental results revealed that among all variants of test specimens, UC1, PM1, and FM1 provided the highest compressive strength. Therefore, the empirical test results and material behavioural curve for these samples have been further analysed to explain their properties by focusing on compressive modulus, yield point, and maximum strength. Figure 9 shows a graphical presentation of those three parameters with grid lines that facilitate the analysis and comparison among the test samples involved.

Before reaching the yield point, every sample shows a linear relationship indicating the elastic deformation where the samples are capable of regaining their original shape upon the elimination of test loads up to this extent. The slope of the linear relationship in Figure 9a describes the degree of material stiffness, also known as compressive modulus. The compressive modulus value is proportional to each sample’s ability to resist deformation (stiffness). According to Figure 9a,b, the slope of the linear curve is higher in FM (7.64 GPa) followed by PM (5.98 GPa) and UC (2.84 GPa). These findings indicate that TSPC is stiffest under full metal tube confinement, stiffer under partial metal tube confinement, and less stiff without any confinement. The bar chart in Figure 9b shows the compressive modulus value increase from UC, PM, and FM. The effect of confinement size may be concluded by increasing the stiffness of the TSPC column specimens.

The linear relationship due to elastic behaviour response as displayed by each test sample (FM, PM, and UC) from the beginning of compressive loading was then changed at one point and this point is known as the yield point. The yield point is identified by observation of noticeable changes in the material behaviour line from linear to nonlinear or from straight to a significant bend that occurs. According to Figure 9a,c, the yield stress value is higher in FM (125.33 MPa) followed by PM (78.58 MPa) and UC (41.41 MPa). These findings indicate that the yield strength of TSPC depends on the percentage of confinement area on TSPC as FM provided the highest yield strength compared to PM. The bar chart in Figure 9c shows the increase in yield stress value from UC, PM, and FM. Both compressive modulus and yield stress value, shown by the experimental results, reveal that the stress transfer from TSPC core to metal tube confinement occurs gradually at the start of compressive loading because the elastic modulus for every sample is different. The rate of stress transfer between TSPC core and confinement was then increased after yield point due to the larger different in yield stress value for every sample.

After yielding, all of the test samples continue to gain strength in plastic straining conditions. The strain hardening behaviour continues until maximum strength is achieved. Upon reaching maximum strength, the material behaviour turns into strain softening up to fracture. According to Figure 9a,d, the compressive strength value indicates that FM has the highest strength (131.84 MPa) followed by PM (95.86 Mpa) compared with unconfined TSPC (59.19 Mpa). The percentage of strength enhancement is 97.78% with the application of fully metal tube confinement (FM sample) and 44.83% with the application of partial metal tube confinement (PM sample). The bar chart in Figure 9d shows the compressive stress value increase from UC, PM, and FM. The effectiveness of metal tube confinement is proven by observing how the amount of metal tube confinement has successfully enhanced the compressive strength of TSPC.

### 3.5. Failure Modes of Unconfined TSPC

Initially, the test sample is subjected to uniaxial compressive force. For unconfined TSPC, the uniaxial force is gradually applied until the sample failed upon maximum principal stress generated through the sample exceeding the material strength. In the case of confined TSPC, the test load is becoming tri-axial force consisting of axial compressive force and lateral force from the confinement material. The tri-axial force acting on the TSPC core has generated combined principle and shear stress. Upon the maximum principle and shear stress generated exceeding the material strength for both TSPC core and confinement material, then failure occurs. The different failure mechanism has produced different failure modes on test samples for unconfined TSPC (UC), partial metal confinement (PM), and full metal confinement (FM).

**Figure 10:**
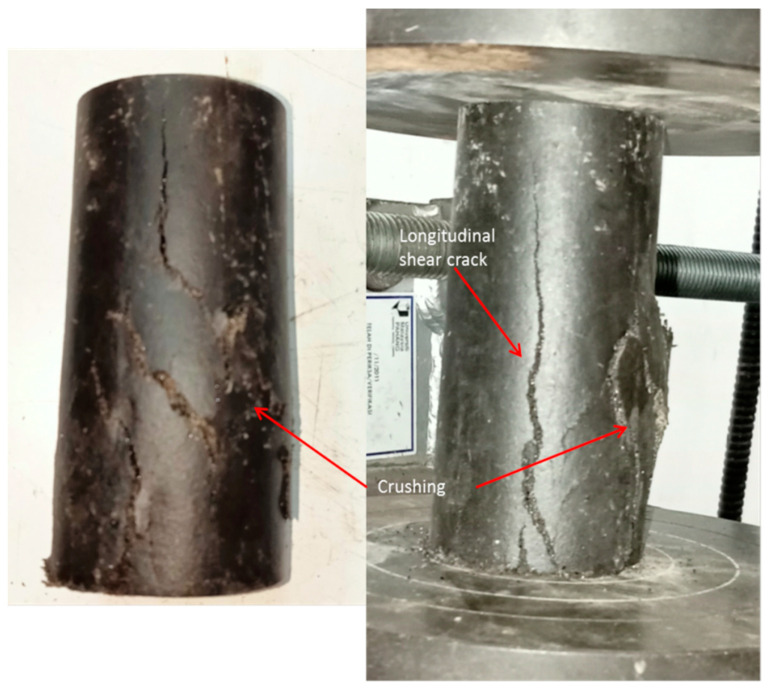
Failure modes of unconfined TSPC (UC).

Figure 10 describes the failure mode of unconfined TSPC as a control sample. From the figure, there was a sign of a longitudinal crack which originated from the bottom of the test sample (UC). This is due to the larger and branched crack formation on the bottom compared to the upper location on the UC test sample. Other than a longitudinal crack, there was also a sign of crushing failure detected on the lower middle section at one side of the unconfined TSPC sample. The combination of cracking and crushing failures may be because even under strain softening fracture, TSPC still holds some strength that prevents it from total rupture. The remaining strength is high enough to induce a secondary type of failure mechanism. From observation, the primary failure that occurs is cracking; before total fracture, the formation of crushing failure has been spotted.

### 3.6. Failure Modes of TSPC with Partial Metal Tube Confinement

**Figure 11:**
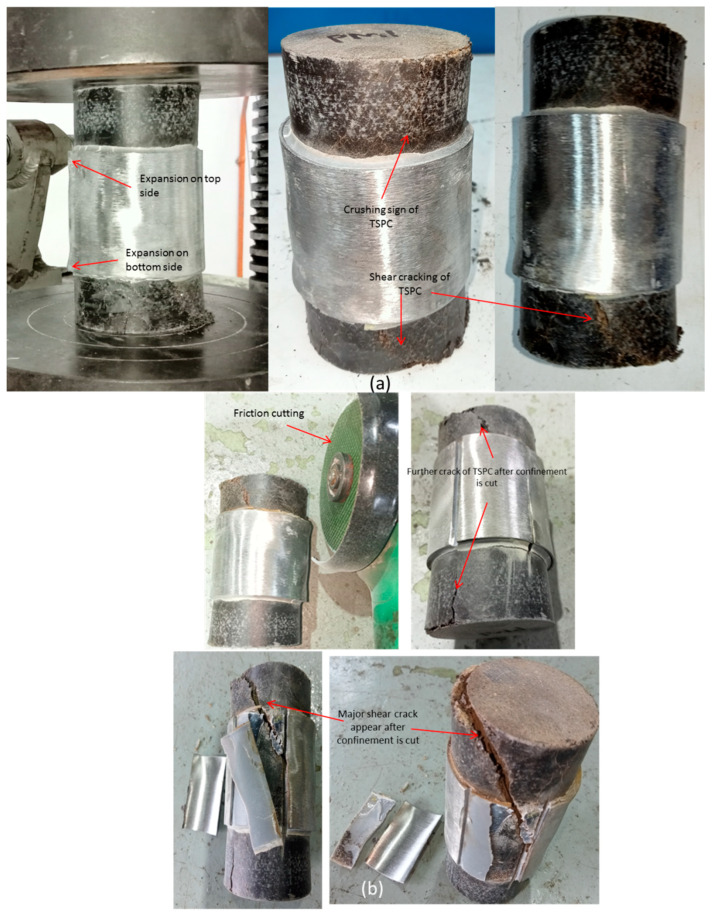
Failure modes of TSPC with partial metal tube confinement. (**a**) PM sample condition after mechanical testing. (**b**) PM sample with bottom side view and the cut open of confinement for failure examination.

Figure 11 explained the failure mode of TSPC with partial metal tube confinement represented by the PM sample. For partial confinement, the initial load is totally endured by the TSPC core because the testing load from the top pressure plate is not touching the confinement at all. Therefore, the primary load is resisted by TSPC, causing lateral expansion in the middle of the TSPC column. Then, due to the confinement effect, the stress transfer occurs from TSPC to confinement and the strength regain has been exhibited due to this condition (resistance to lateral expansion by TSPC core). From Figure 11a, some expansion occurred on both the top and bottom sides of the metal tube confinement due to secondary compressive load resistance. Observation on the TSPC core has shown three types of failure; shear cracking and crushing failure. Another minor failure type that can be observed is longitudinal cracking failure. Shear cracking occurs at the bottom side of the test sample and longitudinal cracking occurs at the top side. Some crushing was detected on the top side and more crushing on the bottom side of the PM sample. A friction cutting is employed to cut open the metal tube to examine the condition inside the confinement. According to Figure 11b upon the cutting of the metal tube, the shear crack and longitudinal crack on the bottom side have progressed further into a bigger opening indicating that the metal tube is holding the TSPC from fracture. Then, after a small portion of the confinement is peeled out, the observation on the adhesive binder indicates that it is in good condition but some debonding has occurred between the confinement and the TSPC core. In addition to that, previously detected shear cracking has also progressed under confinement and with the total removal of that small portion from confinement material the larger opening from the cracking has occurred.

### 3.7. Failure Modes of TSPC with Fully Metal Tube Confinement

**Figure 12:**
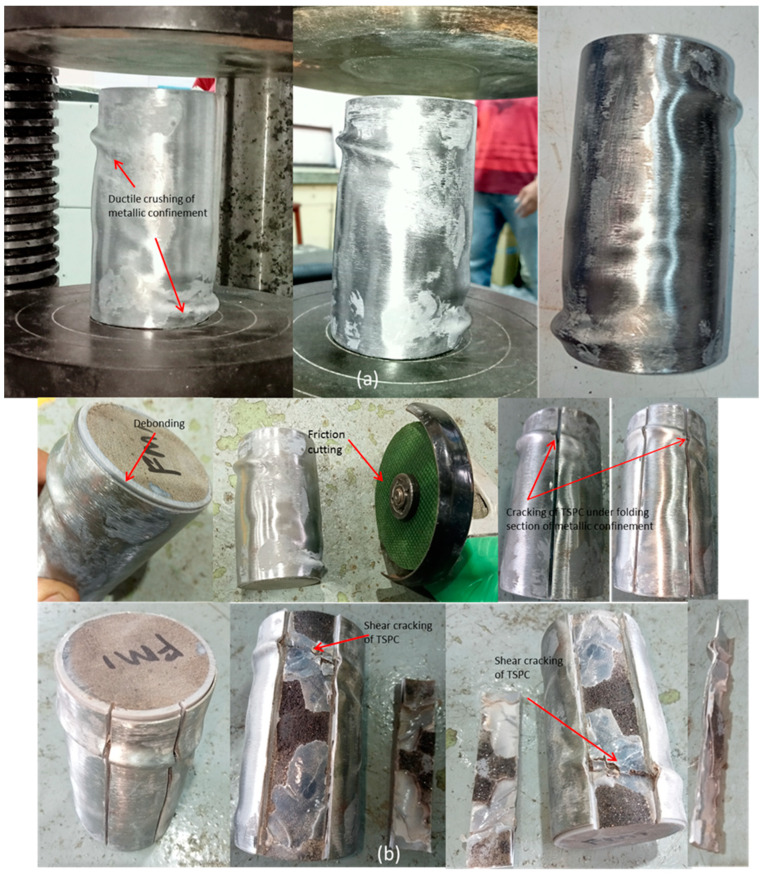
Failure modes of TSPC with fully metal tube confinement. (**a**) FM sample condition after mechanical testing. (**b**) The cut open of metal tube confinement for internal failure examination.

Figure 12 shows the failure mechanism of TSPC with fully metal tube confinement represented by the FM sample. Different from the PM sample, the confinement application has covered the whole TSPC area from bottom to top. This condition has caused the top pressure plate not only to touch the TSPC top surface but also the top surface of the metal tube confinement. Therefore, during test loadings, both the TSPC and metal tube confinement will resist the loading, producing compressive strength values representing the FM sample. Detailed average strength and the test sample behaviour may be referred to in the previous section.

In this section, the failure modes of TSPC with fully metal tube confinement have been observed and examined. Regarding the failure mechanism, Figure 12a shows the FM sample condition after mechanical testing. Due to full metal tube confinement, the condition of the TSPC core is hidden inside and cannot be seen. However, observation of the FM sample condition has revealed a similar general failure mechanism of metallic material which experience ductile failure. The loss in strength occurs after plastic deformation, shown by ductile crushing of the metal tube. The ductile crushing has caused several metallic shells to fold randomly distributed on the whole metal tube confinement material, which is apparent on the top and bottom sides but at different locations indicating the likelihood of twisting of the metal tube confinement.

Intense observation by close-up view reveals that debonding between the TSPC core and metal tube has occurred as shown in Figure 12b. Additionally, in the figure, the FM sample is cut open in small sections by friction cutting to examine what happened to the TSPC core that is initially hidden behind the confinement. The debonding is proven as the cut on a small portion of the metal tube easily peeled out from the FM sample. The observation revealed that debonding occurs randomly where some adhesives are bound to TSPC, and some are bound to the metal tube. In addition, some of the shear cracking failures have also been detected on the TSPC which is not visible before the confinement is cut open. The shear cracking location occurred right behind the metal tube with apparent ductile folding on the top and bottom of the FM sample.

The FM test samples have shown similar failure behaviour with PM sample but with higher magnitude of compressive modulus, yield strength, and maximum strength (Figure 9). Both samples exhibited repeats in the strain hardening response due to the primary loss of strength and regain strength before fracture. The relationship between failure modes on FM sample with its behavioural curve under compression is likely due to premature debonding between TSPC and metal tube confinement. This situation occurs due to the simultaneous compressive load imposed on both the TSPC and metal tube confinement top surface. However, both the top pressure plate and bottom pressure plate of the test machine hold the FM sample to resist further load increments until a clear sign of strength loss due to ductile crushing of the metal tube is detected by the machine actuators and the test is finished. In general, full metal tube confinement has effectively been proven to enhance the TSPC strength to a maximum level but in a particular way. A summary of the failure modes on all samples is listed in Table 4.

## 4. Conclusions

The experimental study on TSPC strengthening under compression with metallic material confinement was performed with metal tube confinement on a TSPC short-column specimen. This study yields 100% novelty considering the metallic material application to enhance TSPC strength through external constraint has never been reported in the previous literature. In this study, the compressive strength of TSPC enhanced with metal tube confinement application from 59.19 MPa (UC1) to 95.86 MPa (PM1) and 131.84 MPa (FM1) representing 61.95% and 122.74% of strength enhancement percentage. The enhancement was measured after a compressive test on TSPC short column with the application of an average 0.5 mm thickness of confinement binders (Epoxy Sikadur 330) and 1 mm thickness of MS metal tube (ASTM 106) with partial (PM) and fully (FM) confinement. For comparison, previous studies on concrete external strengthening by Pannachet and Boonpichetvong [29,30] which employ 0.69 mm thickness of mild steel sheet and 0.75 mm thickness of zinc–alum metal sheet as confinement on the concrete column have provided 98% and 153.1% of strength enhancement. However, without counting the performance of metal confinement on conventional cement concrete strengthening, there was better maximum strength by FRP confinement on TSPC in particular where the maximum percentage of compressive strength enhancement of 128.06% was recorded in the previous study with the application of CFRP confinement [11]. In spite of that, metallic material confinement preferably has revealed different failure behaviour. In general, FRP confinement on TSPC has shown sudden and brittle fracture during failure where the material behavioural curve has no sign of strain softening but with metallic material confinement, the curve has shown a large amount of strain softening before fracture. This behaviour occurs on both TSPC with partial (PM) and full (FM) metallic material confinement. In addition, the failure mode has indicated that the high ductility of metallic material confinement has effectively held the TSPC from sudden fracture where the metal tube in partial confinement shows ductile expansion while the metal tube in full confinement has shown ductile crushing. This failure mode is preferable especially for safety criteria in the design consideration of a structural material because the ductile deformation on metallic material confinement occurs gradually with pre-failure warning signs to provide enough time for hazard management and corrective action. In addition, metal confinement is also capable of absorbing potential failure due to impact and cyclic loading with its ductile behaviour. Generally, this study can conclude that metallic material confinement on TSPC under compressive load has successfully providing the strength enhancement and modified the failure behaviour of TSPC. Further empirical studies on metallic material confinement on TSPC may continue to compare between different types of metallic material confinement or examine the effect of long-term exposure under various conditions on metallic material confinement. In addition to that, a study on the optimal reinforcement method on TSPC can also be carried and obtained through quantitative experimental results analysis in future research. Additionally, numerical analysis using finite element model may also be initiated to provide parametric study on metallic material confinement on TSPC.

## Figures and Tables

**Table 1:** Summary of TSPC compressive strength performance under various graded, composition, and preparation methods.

Study	Aggregates Grading (TS Particles Size)	Matrix Resin	Aggregates to Resin Ratio	Curing Temperature	Curing Time	Compressive Strength (Mpa)
Faidzal et al., 2018 [6]	Uniformly graded—Fine <1 mm	Unsaturated Polyester Resin + 2% Methyl Ethyl Ketone Peroxide (MEKP)	70–30	Room Temperature (23—33 °C)	3 days	58.21
	Uniformly graded—Semi Fine 1 mm		65–35			56.23
	Uniformly graded—2 mm		70–30			43.70
	Uniformly graded—Coarse > 2 mm					30.41
Shakil and Hassan, 2020 [16]	Gap graded—4 mm + 2 mm					37.71
	Gap graded—3 mm + 1.5 mm					35.79
	Gap graded—2 mm + 1 mm					32.87
Hassan et al., 2020 [17]	Uniformly graded—Fine <1 mm				24 h	59.20
Amirnuddin et al., 2021 [19]	Uniformly graded—Fine <1 mm					62.24
Abdullah, 2021 [18]	Uniformly graded—Fine <1 mm					59.20

**Table 2:** Description of test samples and specimen code.

Sample Code	Specimen Number	Confinement Description	Confinement Adhesive Curing Condition
UC	UC1	Control sample (Unconfined)	N/A
	UC2		
	UC3		
PM	PM1	Partial metal confinement 50 mm on middle section	30 days of curing at room temperature (Sikadur 330 manufacturer)
	PM2		
	PM3		
FM	FM1	Full metal confinement 100 mm on TSPC column	
	FM2		
	FM3		

**Table 3:** Summary of mechanical properties of test samples.

Samples	Specimen Designation	Maximum Deformation (mm)	Maximum Load (kN)	Compressive Strength (MPa)	Fractured Energy (J)	Average Strength (MPa)	Percentage of StrengthEnhancement
UC	UC1	3.028	116.22	59.19	354.31	58.37	-
	UC2	3.07	112.59	57.34	387.01		
	UC3	3.028	115.00	58.57	341.39		
PM	PM1	2.614	188.23	95.86	1123.74	84.53	44.83%
	PM2	4.288	155.28	79.08	1080.03		
	PM3	3.458	154.44	78.66	960.23		
FM	FM1	2.872	258.86	131.84	1236.38	115.44	97.78%
	FM2	2.726	216.52	110.27	1405.21		
	FM3	8.986	204.63	104.22	1821.98		

**Table 4:** Summary of failure modes on all test samples.

Sample	Failure Modes	Potential Causes
UC	Longitudinal cracks and crushing	Transverse load generated causes the crack formation and longer strain softening curve produce crushing.
PM	Lateral expansion on top and bottom of metallic confinement material. Shear cracking and crushing on TSPC core.	Secondary compressive load resistance from confinement yield expansion on both ends of confinement material. After that, further stress generated from compressive force exceeded TSPC strength causing crack and crushing failure.
FM	Ductile crushing of the metallic confinement material. Shear crack on TSPC core behind the folding area of the metallic confinement material.	Ductile crushing occurs due to loss in strength of metallic confinement material after plastic deformation. Then, load generated from compression after confinement failure exceeded load carrying capacity of TSPC causing shear cracks.

## Data Availability

Data are unavailable due to privacy or ethical restrictions.

## References

[1] Mebarkia S., Vipulanandan C. Aggregates, Fibers and Coupling Agent in Polyester PC. Proceedings of the Materials Engineering Congress, ASCE.

[2] Pyataev E., Zhukov A., Vako K., Burtseva M., Mednikova E., Prusakova M., Izumova E. (2019). Effective polymer concrete on waste concrete production. E3S Web Conf..

[3] Wang G.C. (2016). Slag use as an Aggregate in Concrete and Cement-based Materials. The Utilization of Slag in Civil Infrastructure Construction.

[4] Singh G., Kansal H., Khangura S., Singh P., Singh H., Brar G. (2014). Polymer Concrete Composites Made from Industrial Waste Materials: A Review. Proceedings of the International Conference on Research and Innovations in Mechanical Engineering: ICRIME-2013.

[5] Ismail S., Hoe K.W., Ramli M. (2013). Sustainable aggregates: The potential and Challenge for Natural Resources Conservation. Procedia Soc. Behav. Sci..

[6] Faidzal M.M.Y., Hassan S.A., Omar B., Zakaria K., Zaharuddin M.F.A. (2018). Particle Size Effect on Optimal Mixture Ratio of Tin Slag Polymer Concrete under Compression. J. Built Environ. Technol. Eng..

[7] Manda M., Rejab M., Hassan S., Ma Q., Hassan R., Hamid N.H.A., Arshad A.K., Alisibramulisi A., Sidek M.N.M., Bhkari N.M., Shaffie E. (2022). A Review on Tin Slag Polymer Concrete as Green Structural Material for Sustainable Future. Green Infrastructure: Materials and Applications.

[8] Bedi R., Chandra R., Singh S.P. (2014). Reviewing some properties of polymer concrete. Indian Concr. J..

[9] Asif A., Ansari A. Polymer Concrete as Innovative Material for Development of Sustainable Architecture. Proceedings of the 2nd International Conference on Emerging Trends in Engineering & Technology.

[10] Yeon K.S. (2010). Polymer Concrete as Construction Materials. Int. J. Soc. Mater. Eng. Resour..

[11] Langer W.H., Arbogast B.F. (2002). Environmental Impacts of Mining Natural Aggregate. Deposit and Geoenvironmental Models for Resource Exploitation and Environmental Security.

[12] Ozcan O., Musaoglu N., Seker D.Z. (2012). Environmental Impact Analysis of Quarrying Activities Established on and near a River Bed by Using Remotely Sensed Data. Fresenius Environ. Bull..

[13] Ukpong E.C. (2012). Environmental Impact of Aggregate Mining by Crush Rock Industries in Akamkpa Local Government Area of Cross River State. Niger. J. Technol..

[14] Raza S., Khan M.K.I., Menegon S.J., Tsang H.H., Wilson J.L. (2019). Strengthening and Repair of Reinforced Concrete Columns by Jacketing: State-of-the-Art Review. J. Sustain..

[15] Ueda T. Material conditions necessary for strengthening concrete structures. Proceedings of the 2nd International Conference on Sustainable Civil Engineering Structures and Construction Materials 2014 (SCESCM 2014).

[16] Shakil U.A., Hassan S.A. (2020). Behavior and properties of Tin Slag Polyester Polymer Concrete Confined with FRP Composites under Compression. J. Mech. Behav. Mater..

[17] Hassan S.A., Hanan U.A., Yahya M.Y., Wahit M.U. (2020). Behaviour of Tin Slag Polymer Concrete Confined with Carbon Fibre Reinforced Epoxy. J. Penelit. Karya Ilm. Lemb. Penelit. Univ. Trisakti.

[18] Abdullah K.F. (2021). Compressive Behavior of Tin Slag Polymer Concrete Confined with Fiber Reinforced Polymer Composites Exposed to Tropical Weathering and Aggressive Conditions. Bachelor’s Degree Thesis.

[19] Amirnuddin S.S., Hassan S.A., Wahit M.U., Rejab M.R.M., Basheer U.M., Khalid N.H.A. Compressive Properties and Behaviour of Tin Slag Polymer Concrete Exposed to Tropical Climate and Aggressive Environment. Proceedings of the 2nd International Professional Doctorate and Postgraduate Symposium 2021 (iPDOCs’21).

[20] Domone P., Illston J. (2010). Strength and failure of concrete. Construction Materials.

[21] Chin C.L., Ma C.K., Tan J.Y., Ong C.B., Awang A.Z., Omar W. (2019). Review on development of external steel-confined concrete. J. Constr. Build. Mater..

[22] Zhou X.H., Liu J.P. (2010). Seismic behavior and shear strength of tubed RC short columns. J. Constr. Steel Res..

[23] Kedziora S., Anwaar M.O. (2019). Concrete-Filled Steel Tubular (CFTS) Columns Subjected to Eccentric Compressive Load. AIP Conf. Proc..

[24] (2019). Standard Practice for Making and Curing Concrete Test Specimens in the Laboratory.

[25] (2019). Molds for Forming Concrete Test Cylinders Vertically.

[26] Umamaheswari N., Jayachandran S.A. (2014). Influence of Concrete Confinement on Axial Load Capacity of Concrete-filled Steel Tubes. J. Civ. Eng. Res..

[27] (2008). Guide for the Design and Construction of Externally Bonded Confinement Systems for Strengthening Concrete Structures.

[28] (2001). Standard Test Methods for Compressive Strength of Chemical-Resistant Mortars, Grouts, Monolithic Surfacings and Polymer Concretes.

[29] Pannachet T., Boonpichetvong M. (2018). Numerical Investigation of Axial Strength Development in Metal Sheet Confined Concrete. Eng. J. Res. Dev..

[30] Pannachet T., Boonpichetvong M. (2018). Axial Compressive Strength of Metal Sheet Confined Concrete Cylinders Based on Various Concrete Strengths. Civ. Eng. J..

